# Relative Roles of Weather Variables and Change in Human Population in Malaria: Comparison over Different States of India

**DOI:** 10.1371/journal.pone.0099867

**Published:** 2014-06-27

**Authors:** Prashant Goswami, Upadhayula Suryanarayana Murty, Srinivasa Rao Mutheneni, Swathi Trithala Krishnan

**Affiliations:** 1 CSIR Fourth Paradigm Institute, Bengaluru, India; 2 CSIR Indian Institute of Chemical Technology, Hyderabad, India; Swiss Tropical & Public Health Institute, Switzerland

## Abstract

**Background:**

Pro-active and effective control as well as quantitative assessment of impact of climate change on malaria requires identification of the major drivers of the epidemic. Malaria depends on vector abundance which, in turn, depends on a combination of weather variables. However, there remain several gaps in our understanding and assessment of malaria in a changing climate. Most of the studies have considered weekly or even monthly mean values of weather variables, while the malaria vector is sensitive to daily variations. Secondly, rarely all the relevant meteorological variables have been considered together. An important question is the relative roles of weather variables (vector abundance) and change in host (human) population, in the change in disease load.

**Method:**

We consider the 28 states of India, characterized by diverse climatic zones and changing population as well as complex variability in malaria, as a natural test bed. An annual vector load for each of the 28 states is defined based on the number of vector genesis days computed using daily values of temperature, rainfall and humidity from NCEP daily Reanalysis; a prediction of potential malaria load is defined by taking into consideration changes in the human population and compared with the reported number of malaria cases.

**Results:**

For most states, the number of malaria cases is very well correlated with the vector load calculated with the combined conditions of daily values of temperature, rainfall and humidity; no single weather variable has any significant association with the observed disease prevalence.

**Conclusion:**

The association between vector-load and daily values of weather variables is robust and holds for different climatic regions (states of India). Thus use of all the three weather variables provides a reliable means of pro-active and efficient vector sanitation and control as well as assessment of impact of climate change on malaria.

## Introduction

A topic of growing concern, and debate, is the role of climate change in variability and trends in malaria [1]–[5]. Several countries and regions have reported emerging or growing threat of malaria due to change in climate [6]–[10]. While deaths due to malaria depend on a number of factors [11]–[13] like vector control, access to adequate medical facilities and immunological history of the people, infections can be considered to depend primarily on abundance of mosquito vector and exposure of the host to bites and rate of transmission. However, for a population with given immunological history, transmission can be assumed to be constant, at least at short time scales (∼ years). Thus the abundance of vectors and the degree of exposure can be said to determine the vulnerability and the severity of the epidemic. However, in regions of rapidly changing human populations, like in many states of India, the relative roles of abundance and change in exposure due to changing human population are not easy to determine. A quantitative delineation of the role of weather variables in malaria load can enable design of pro-active mitigation based on vector control; implementation of such pro-active vector control is becoming increasingly important in view of reported resistance of mosquitoes to insecticides like DDT [14]. At the same time, such quantitative relations between weather variables and malaria can enable reliable projection of vulnerability in a changed climate.

The importance of meteorological variables in genesis and survival of mosquito have been known for a long time, although the relative roles of these variables and various aspects are still being investigated. However, fairly sharp and well defined ranges of meteorological variables, especially temperature, are required for genesis and survival of malaria vector, although these thresholds also depend on the mosquito species [15]–[19]. For example, there are well known thresholds of minimum and maximum of temperature for vector genesis and survival. Each weather variable has a range in which it affects the mosquito genesis and survival; a temperature above 16°C is required for breeding and development of the aquatic stages of Anopheline mosquitoes in the Tropics. Similarly, experiments indicate a threshold of minimum temperature between 16°C and 19°C for the development of *Plasmodium falciparum* (PF) malaria parasite. Temperature affects the rate of reproduction of the pathogens, and thus plays important role in the survival and transmission the vector [20]. Temperature is also known to affect the gonotrophic cycle (physiological process consisting of digestion of blood-meal and development of ovaries), and longevity. However, other weather variables like rainfall and humidity also play important roles [20]–[21]. For example, amount, intensity as well as duration of rainfall all play important roles in the dynamics of vector population. Besides, other environmental variables like land use and land cover also affect vector dynamics [22]–[23]. Rainfall can significantly reduce the vector survival by flushing the larval mosquitoes out of their aquatic habitat [17]; it can be logically assumed that reduced larval survival will also lead to a smaller population of adult mosquitoes. Both dryness and rainfall can influence larval survival significantly, as shown by experiments with simulated dry conditions [16]–[17]. Similarly, transmission can be inhibited by development time of the pathogen larger than the life span of the insect [18]. Higher temperature, on the other hand, can lead to quicker maturity leading to higher number of off springs during the transmission period [19]. Thus vector abundance can be assumed to be primarily determined by the climate (average weather regime) and the weather (variability around the climate) of the region [15]–[22].

It is also known that the effect of environmental parameters on vector population can be highly location-specific. The phenology and population dynamics of mosquito can vary significantly with altitude; a study based on weekly rainfall intensity in the Lake Victoria basin showed significant differences in larval abundance with altitudes [24]. It was found, for example, that for northern Australia, determination of the periodicity and the amplitude of mosquito population (abundance peaks) required consideration of the frequency and the amplitude of the tides [25]; a flood plain or a forested region is likely to require very different considerations. Over India, several studies have shown relationship between weather variables and malaria [26]–[28]. India with its complex climatic heterogeneity, growing population, land-use and urbanization poses a unique challenge to identify role of climate change in malaria load [29]–[31]. In particular, a region can experience both decrease and increase in malaria prevalence as the meteorological variables move in or out of the genesis window. A robust and reliable assessment of vulnerability to malaria due to climate change require quantitative, validated models of malaria epidemiology that involve all the relevant weather variables; assessments based on the trend of a single meteorological variable do not have much relevance. While the role of meteorological variables in malaria has been well recognized, and several attempts have been already made to use weather-driven models for malaria [32]–[33], most methods have used only a single meteorological variable or time-averaged (weekly/monthly mean) values to assess impact of climate change on malaria. However, dynamics of mosquito population (genesis as well as mortality) depends on daily variability of the weather variables [19]; in general, this daily variability will be much higher than that in monthly-averaged variables. Thus, vector abundance, and hence impact of malaria, is likely to depend on the number of daily genesis windows.

There have been attempts to quantitatively describe the prevalence of malaria relating entomological parameters to malaria transmission that include many parameters like age-at-infection, human blood index, entomological inclusion rate, vectorial capacity etc. However, most of these attempts often do not involve all the weather variables explicitly, and may involve climate variables only indirectly [34] A critical gap is the relative roles of the three meteorological variables (temperature, rainfall, humidity) in relation to the number malaria cases. Although models of population dynamics of vector that consider effects of variables like temperature and rainfall have been considered [35]–[36], they often lack validation against observed epidemiological data. A model of malaria based on weather and exposure has been validated against the observed malarial cases over Arunachal Pradesh in north-east India [37]. However, there are several aspects that were neither covered nor inferable from our previous study [37]. An important issue is the robustness of the results in terms of applicability to diverse climatic regions. This issue is addressed in the present manuscript by considering climatically and endemically diverse regions in terms of the 28 states of India; these states represent climates from the tropical to the higher latitudes. Another important issue is the delineation of the role of weather variables from those of other socio-economic variables; this issue is addressed in the present work by considering the impact of human population. In particular, analysis with constant and varying populations are carried out to quantify the impact of change in the number of hosts (as against change in vector population due to weather variables) in the disease load. While impact of human population may be minimal in regions like Arunachal Pradesh, the change (increase) in exposure due to growth in human population can be appreciable in many areas of India. Similarly, quantification of the roles of the three weather variables is expected to fill a critical gap in our understanding of the dynamics of malaria.

## Methods

### Ethics Statement

We declare that the data on malaria cases in this study was collected and compiled by the co-authors from CSIR-IICT based on records at the Public Health centres.

The collected epidemiological data from primary health centers was analyzed anonymously and no particular patient by name was involved. The study received clearance from the institutional Ethical Committee at CSIR-Indian Institute of Chemical Technology.

### Study Area

We consider the 28 states of India, characterized by wide ranges of temperature, humidity and rainfall (Table 1). To apply our analysis to the 28 states, we use the annual malarial case data from the state health directorate. The population data was adopted from www.faostat.fao.org.

**Table 1:** Showing Abbreviations used for State name, annual (1961–2010) mean minimum and maximum values of temperature, humidity and rainfall averaged over each state.

S.No	Abbreviations	States	Temperature (°C)		Humidity (%)		Rainfall (mm)
			Min	Max	Min	Max	Max
1	AP	Andra Pradesh	23	38	33	90	13
2	Ar P	Aruachal pradesh	12	30	60	88	27
3	AM	Assam	14	32	47	99	20
4	BR	Bihar	15	34	32	99	16
5	DL	Delhi	4	36	48	91	19
6	GA	Goa	18	37	53	97	18
7	GJ	Gujarat	19	34	30	92	11
8	HY	Haryana	3	31	38	91	23
9	HP	Himachal pradesh	7	29	70	96	16
10	JK	Jammu & kashmir	2	28	53	94	16
11	JH	Jarkhand	11	31	44	93	16
12	KA	Karnataka	16	34	54	96	13
13	KL	Kerala	20	35	31	91	27
14	MP	Madhya Pradesh	19	32	49	90	20
15	MH	Maharashtra	20	36	59	97	16
16	MN	Manipur	14	30	43	96	19
17	MG	Meghalaya	16	32	47	92	18
18	MZ	Mizoram	14	30	52	98	11
19	NG	Nagaland	16	29	30	93	23
20	OR	Orissa	14	32	38	91	16
21	PB	Punjab	12	34	70	97	16
22	RJ	Rajasthan	20	39	53	94	6
23	SK	Sikkim	18	31	41	93	13
24	TN	Tamil nadu	21	37	55	93	21
25	TP	Tripura	14	34	33	93	16
26	UP	Uttar Pradesh	8	35	45	91	17
27	UK	Uttar Khand	9	33	57	95	17
28	WB	West Bengal	13	34	45	96	14

### Collection of Malaria Data

The data was collected from the Directorate of Health, State Govts of India, based on cases reported from Primary Health Centers (PHCs) from all the states containing data on epidemiological aspects of Malaria number of blood samples collected (NBSC)and the number of blood samples that tested positive (NBSP) for either *Plasmodium vivax* (NPV) or *Plasmodium falciparum* (NPF) infection, or mixed infection; thus blood sample positive (NBSP) is the sum total of NPV, NPF and mixed type thus provides the total cases of Malaria. The procedure for collecting epidemiological data has been already described in an earlier work [37]; in particular, the data did not involve any personal data or identification of the individuals.

The Epidemiological data on malaria is available online in the National Vector Borne Disease Control Programme(NVBDCP), Ministry of Health & Family Welfare, Govt. of India, (URL: www.nvbdcp.gov.in) and also at (http://nvbdcp.gov.in/Doc/mal-situation-Oct13.pdf).

### Thresholds for vector genesis

The threshold values of meteorological variables were adopted from published literature. Although there have been reports of association between malaria incidence and variables like rainfall with large time lags like 2–3 months such long-term associations are not consistent with the time scales of mosquito life cycles. In our scenario, mosquito genesis takes place whenever favorable meteorological conditions occur; while this time scale in practice could be in hours, we consider daily variables for our description. Each episode of mosquito genesis at daily scale leads to a vector load through the process of survival in terms of biting mosquitoes; this is turn leads to malaria load at daily scale. The annual load of malaria is a sum of these daily values.

We first examine the relation between the annual vector loads, calculated as vector abundance based on the number of genesis days, to determine the relative roles of the meteorological variables. Next we quantify the relative roles of the weather variables (without the effect of size of the human population) and the potential epidemiology (with effect of size human population) and compare against the observed epidemiology.

### Meteorological Data

A critical requirement for our analysis is a long-period, homogeneous data on all the three meteorological variables (temperature, rainfall and humidity) at daily (or shorter) time scale. In the present case these daily values of near surface temperature and near surface humidity were obtained from global NCEP Reanalysis data in a 2.5°*2.5° grid and the daily rainfall on a global 1.8° *1.8° grid [38], [39]. The daily data at state level was created through appropriate interpolation or averaging. The advantage of NCEP Reanalysis is that it provides a comprehensive and consistent long-period data for all the three variables on a grid. It has been also shown that the interpolated NCEP Reanalysis possesses good correspondence with high resolution data from other sources over the region [40], and provides an effective data set for studying role of weather variables in malaria. Information on NCEP data is available on http://www.ncep.noaa.gov/

### Days of Vector Genesis

We define a (vector) genesis day as one on which all the three variables: temperature, rainfall and humidity, are within their respective ranges for genesis of mosquitoes. In particular, a day is counted as a genesis day if
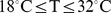


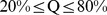


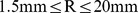
where T, Q and R represent, respectively, daily average temperature, daily average humidity and daily rainfall, extracted from daily gridded data from NCEP Reanalysis.

As vector dynamics takes place on a daily (or even shorter) time scales, we have considered daily values of weather variables in our analysis; the annual values are thus sum over these daily values. It would be ideal, and interesting, to compare the daily values of observed and computed malaria cases. However, as the current data availability on the number of malaria cases is at annual scale, the validation is carried out at annual scale; besides, analysis of trends due to change in climate and population (hosts) is really meaningful only with annual values.

Infection depends both on the number of encounters between human and mosquito (exposure) leading to bites as well as transmission of the parasite. The exposure is a complex function of life style, migration and movement of the human population as well as vector abundance. Naturally, only a fraction of the total human population present is eventually bitten by mosquito; however, it is expected that a larger vector density provides higher exposure leading to more bites. Assuming that larger genesis days allow larger prevalence of vectors, we consider the number of genesis days in a year as representative of vector abundance or vector load. To delineate the role of vector genesis days (and hence climate change) and change of host population, we compute two quantities, as defined below:

The annual vector load (E_V_) for each state for year n is calculated as the sum of the days, N_VG_(n), in the year that satisfy criteria for genesis of mosquitoes.
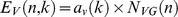
Thus E_V_ represents the number of vector genesis days with a constant host (human) population for the state k. It is assumed that the actual vector load will be a result of modulation of the number of vector genesis days by other parameters like land use and land type not explicitly included here; this modulation for a state is represented through the coefficient α_v_(k) characteristic of the state.

The annual potential malaria load is calculated by including the effect of human population as
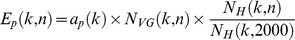
Here N_H_(k,n) is the human population for the state k in the year n. The coefficient α_v_(k) and α_p_(k) characterize our model in terms of modulation of vector load and malaria load through processes not explicitly included. For example, α_p_(k) can contain information specific to the state like immunological history (transmission coefficient), and land use. Since only a fraction of the bites will result in infection these coefficients generally would have values ≪1, depending on the number of mosquitoes that carry malaria parasites and encounter human host. However, to isolate the effects of vector genesis we use a single value (0.007) of α_v_(k) as a scaling factor to focus on the role of vector load; thus effects of variation due to exposure and transmission from state to state are not considered here. Similarly, a single value of (0.002) is used for α_p_(k); the difference in the values of α_v_(k) and α_p_(k) is to ensure that the actual values of E_v_ and E_p_ are of similar magnitudes. These values of α_v_(k) and α_p_(k) were obtained through an iterative process to arrive at minimum differences between recorded malaria cases (E_O_(k,n)) and calculated values of malaria for one year and kept fixed for the other years [37]. In other words, the values of α_v_ and α_v_ represent optimum values to obtain best fit between the simulated and the observed values in a statistical sense.

## Results

**Figure 1:**
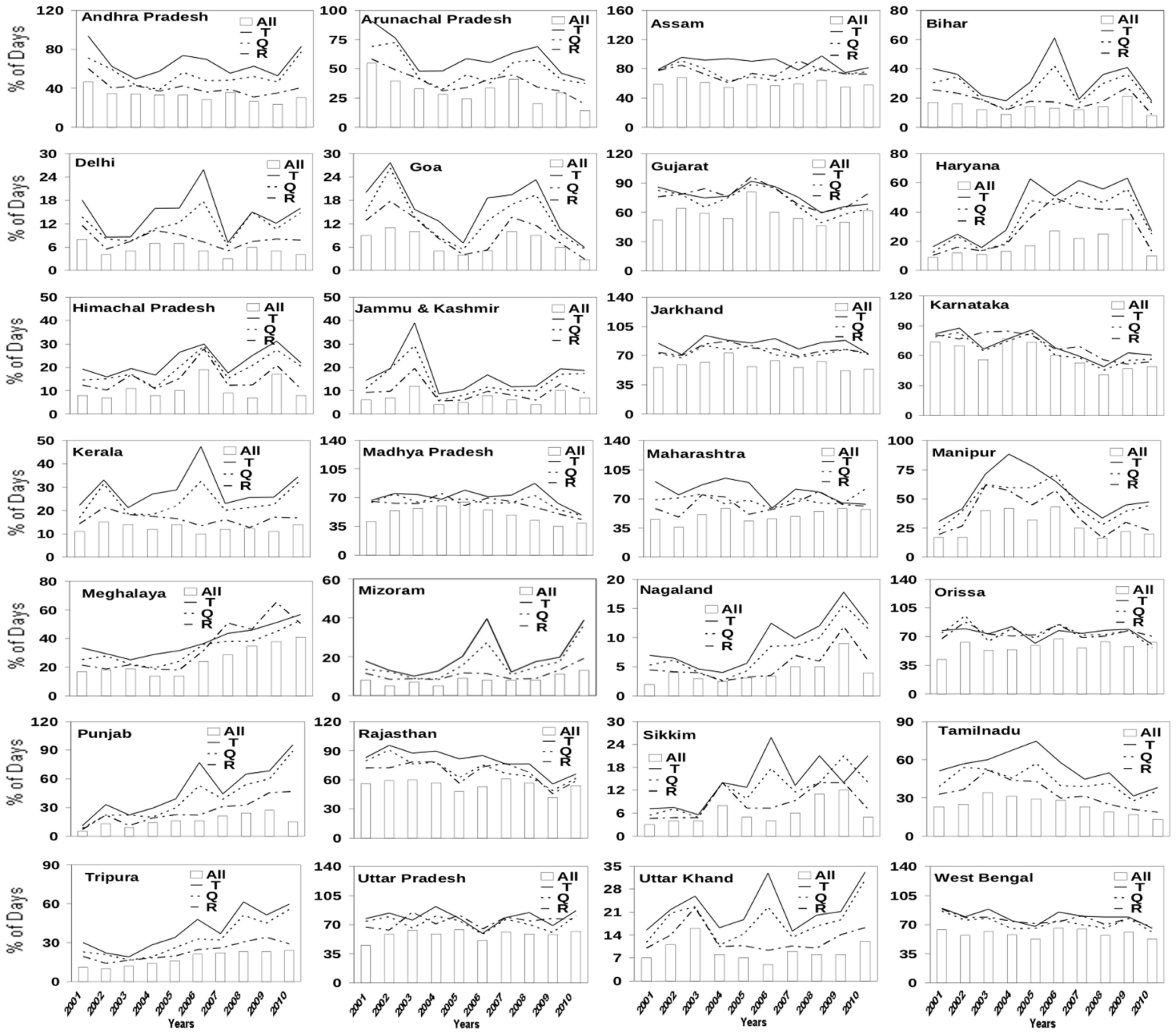
Comparison of % of days that satisfies criteria for vector genesis in terms of individual meteorological parameters like temperature (T), humidity (Q) and rainfall (R) and all the three parameters combined over 28 states of India for the period 2001–2010.

The 28 states of India (Table 1) show substantial variations in variables like annual maximum and minimum temperature. The number (in %) of days satisfying genesis criteria, in terms of all the three weather variables can be very different from those based on a single variable. Indeed, percentages of genesis days based on individual weather variables (T, Q or R) show significant differences not only in their values but also in their variability; the percentage of days based on all the three variables is significantly smaller than that for any single variable, as expected. The inter-annual variability in the % of genesis days with all the three meteorological variables is also very different from the corresponding variability with only one meteorological variable (Figure 1).

**Figure 2:**
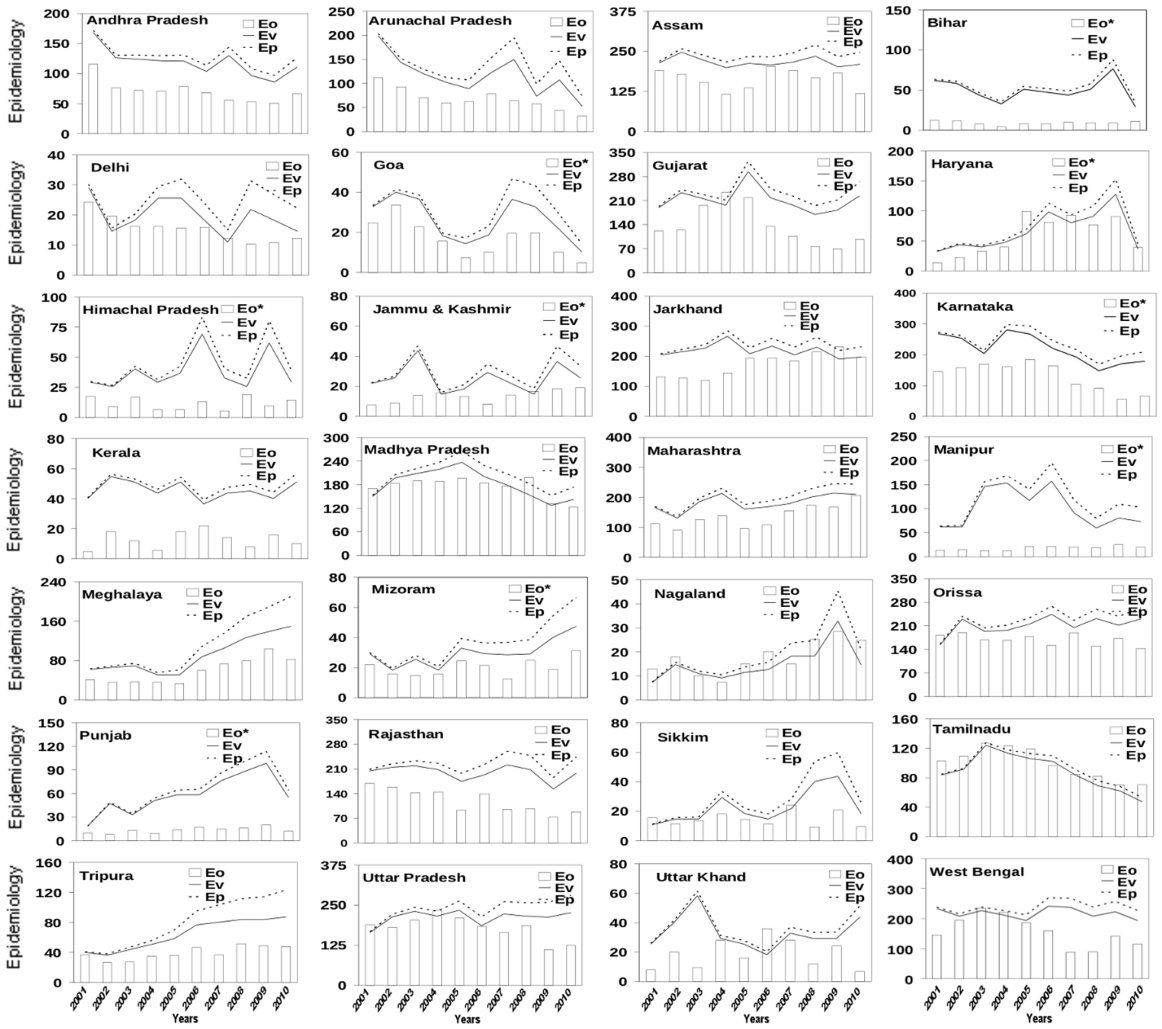
Comparison of observed annual epidemiology load (E_O_) with epidemiology load based on vector load (E_V_) calculated using days of vector genesis and constant human population with potential epidemiology load (E_P_) (growth in human population included) over 28 states of India. The epidemiology is calculated as the number of blood samples that tested positive. The days of vector genesis here represent days in a year that fulfill combined meteorological conditions of temperature, humidity and rainfall for genesis of mosquitoes. The calculated epidemiology has been scaled by a factor (500 for which marked * and rest with 1000, as indicated) for easy comparison.

A comparison of vector load, with vector abundance derived from all the three weather variables, shows excellent agreement (Figure 2) for most of the states, both with and without modulation by growth in human population (E_P_ and E_V_, respectively). As expected from the linear growth in human population in many states, the potential malaria load is always larger than the vector load, with difference between the two generally increasing with time (Figure 2) due to normalization with respect to the population of 2001–2010. However, while correlation between Eo and E_P_ is generally better than that between Eo and E_V_ (Table 2), the effect is not really appreciable; in particular, against 21 states with correlation coefficient equal to or more than 0.8 for predicted potential malaria load the corresponding number for vector load is 18 (Table 2).

**Table 2:** Correlation between observed (E_O_) and estimated (E_P_ and E_V_) epidemiology load with different combination of meteorological variables.

S.No	States	Correlation coefficient between epidemiology load and							
		All		T		Q		R	
		Ev	Ep	Ev	Ep	Ev	Ep	Ev	Ep
1	Andra Pradesh	**0.9**	**0.9**	**0.7**	**0.7**	**0.4**	**0.5**	**0.9**	**0.9**
2	Aruachal pradesh	**0.9**	**0.9**	**0.7**	**0.8**	**0.4**	**0.4**	**0.8**	**0.8**
3	Assam	0.3	**0.6**	0.3	0.2	0.2	0.1	**0.4**	**0.5**
4	Bihar	**0.7**	**0.8**	0.1	0.2	0.3	0.3	**0.4**	**0.4**
5	Delhi	**0.9**	**0.5**	0.2	0.3	0.3	0.3	**0.5**	**0.5**
6	Goa	**0.9**	**0.9**	**0.7**	**0.9**	**0.8**	**0.9**	**0.9**	1.0
7	Gujarat	0.3	**0.5**	**0.5**	**0.6**	**0.5**	**0.6**	**0.7**	**0.7**
8	Haryana	**0.9**	**0.9**	**0.9**	**0.9**	**0.9**	**0.9**	**0.9**	**0.9**
9	Himachal pradesh	**0.7**	**0.8**	0.1	0.1	0.1	0.1	0.1	0.1
10	Jammu & kashmir	**0.9**	**0.9**	0.3	0.2	**0.4**	**0.4**	**0.4**	**0.4**
11	Jharkhand	0.1	**0.6**	**0.5**	**0.4**	0.3	0.3	**0.4**	**0.4**
12	Karnataka	**0.9**	**0.9**	**0.6**	**0.7**	**0.7**	**0.7**	**0.9**	**0.9**
13	Kerala	**0.9**	**0.9**	**0.6**	**0.6**	**0.6**	**0.6**	0.2	0.2
14	Madhya Pradesh	**0.7**	**0.9**	**0.8**	**0.9**	**0.8**	**0.8**	**0.5**	**0.5**
15	Maharashtra	**0.9**	**0.9**	0.3	**0.4**	**0.6**	**0.6**	**0.9**	**0.8**
16	Manipur	**0.4**	**0.5**	0.2	0.2	0.3	0.2	0.2	0.3
17	Meghalaya	**0.9**	**0.8**	**0.9**	**0.9**	**0.9**	**0.9**	1.0	1.0
18	Mizoram	**0.9**	**0.9**	**0.7**	**0.7**	**0.8**	**0.8**	**0.8**	**0.8**
19	Nagaland	**0.8**	**0.7**	**0.9**	**0.9**	**0.9**	**0.9**	**0.8**	**0.7**
20	Orissa	**0.8**	**0.8**	0.2	0.2	**0.4**	**0.4**	0.2	0.2
21	Punjab	**0.9**	**0.9**	**0.6**	**0.6**	**0.6**	**0.6**	**0.6**	**0.6**
22	Rajasthan	**0.7**	**0.8**	**0.6**	**0.8**	**0.8**	**0.8**	**0.4**	**0.4**
23	Sikkim	**0.9**	**0.8**	0.0	0.0	0.0	0.0	**0.4**	**0.4**
24	Tamil nadu	**0.9**	**0.9**	0.5	**0.5**	**0.8**	**0.7**	**0.6**	**0.6**
25	Tripura	**0.9**	**0.9**	**0.9**	1.0	**0.9**	**0.9**	**0.9**	**0.9**
26	Uttar Pradesh	**0.6**	**0.7**	0.1	0.3	0.2	0.2	0.1	0.1
27	Uttar Khand	**0.9**	**0.9**	0.0	0.0	0.2	0.2	0.1	0.1
28	West Bengal	**0.7**	**0.9**	0.2	0.1	0.1	0.2	0.1	0.1
	No of states with correlation coefficient above 95% significance	25	28	15	16	18	18	20	19

Correlation coefficients of 0.8 are above 9.5% level of significance for the degrees of freedom The cases with correlation above 95% level of significance for the degrees of freedom involved are in bold.

**Figure 3:**
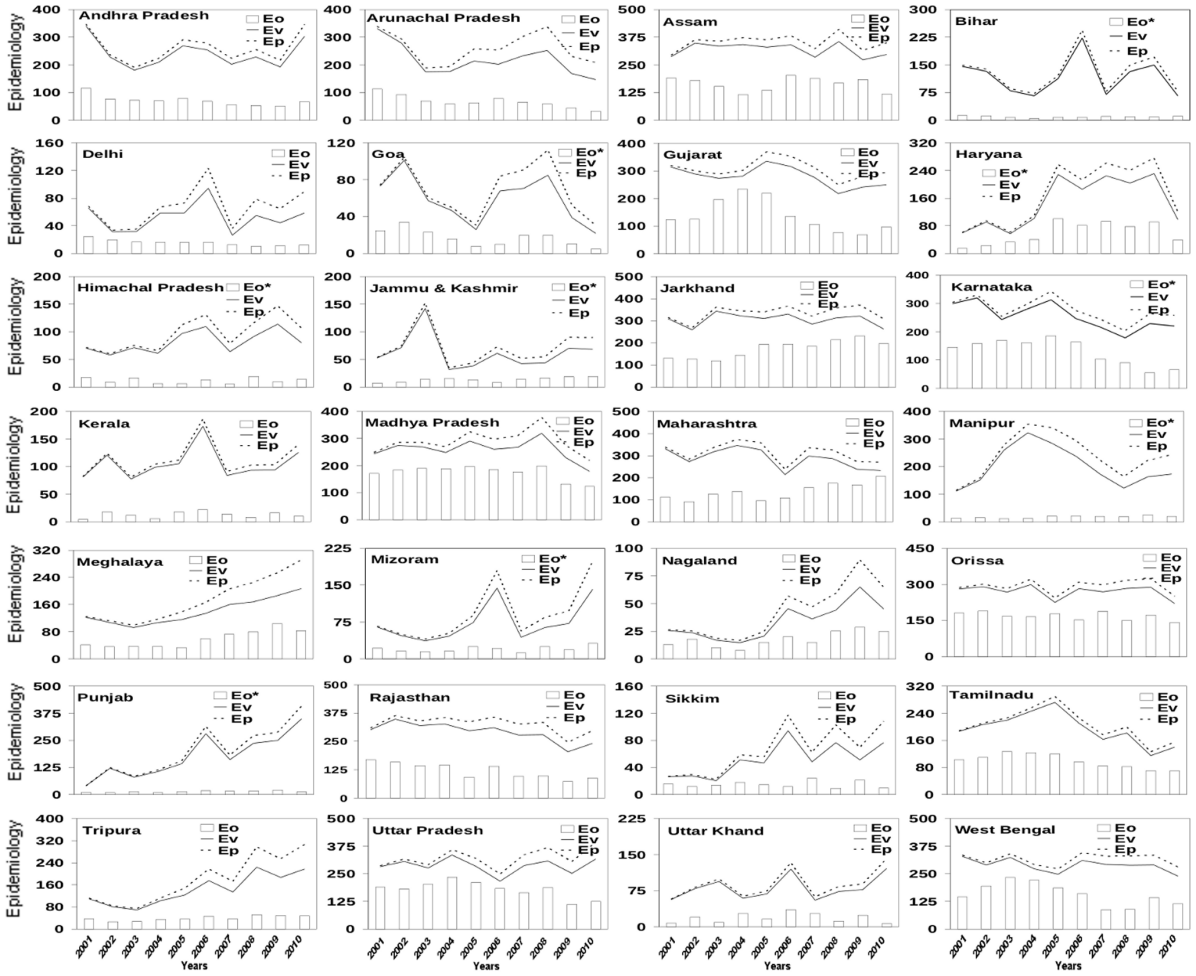
Comparison of observed annual epidemiology load (E_O_) with epidemiology load based on vector load (E_V_) calculated using days of vector genesis and constant human population with potential epidemiology load (E_P_) (growth in human population included) over 28 states of India. The annual epidemiology is calculated as the number of blood samples that test positive. The days of vector genesis represent days in a year that fulfill only meteorological condition of temperature for genesis of mosquitoes. With only temperature as the condition for mosquito genesis, the calculated E_P_ has very little correspondence to the observed E_P_ (Figure 1); only a few (5–8) states show significant correlation (Table 2). The annual epidemiology has been scaled by a factor (500 for which marked * and rest with 1000, as indicated) for easy comparison.

**Figure 4:**
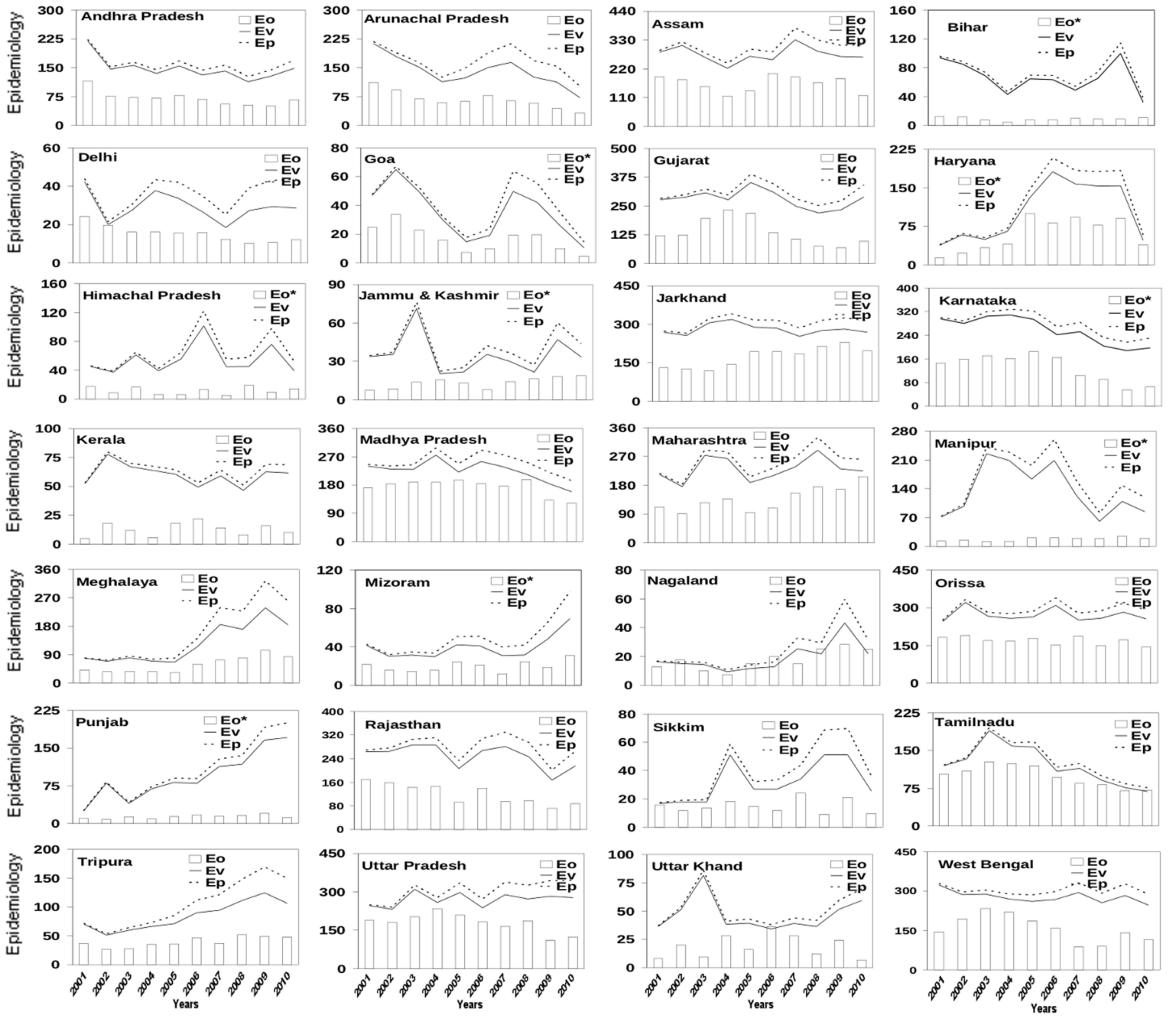
Comparison of observed annual epidemiology load (E_O_) with epidemiology load based on vector load (E_V_) calculated using days of vector genesis and constant human population with potential epidemiology load (E_P_) (growth in human population included) over 28 states of India. The annual epidemiology is calculated as the number of blood samples that positive. The days of vector genesis here represent days in a year that fulfill only meteorological condition of rainfall for genesis of mosquitoes. The annual epidemiology has been scaled by a factor (500 for which marked * and rest with 1000, as indicated) for easy comparison.

**Figure 5:**
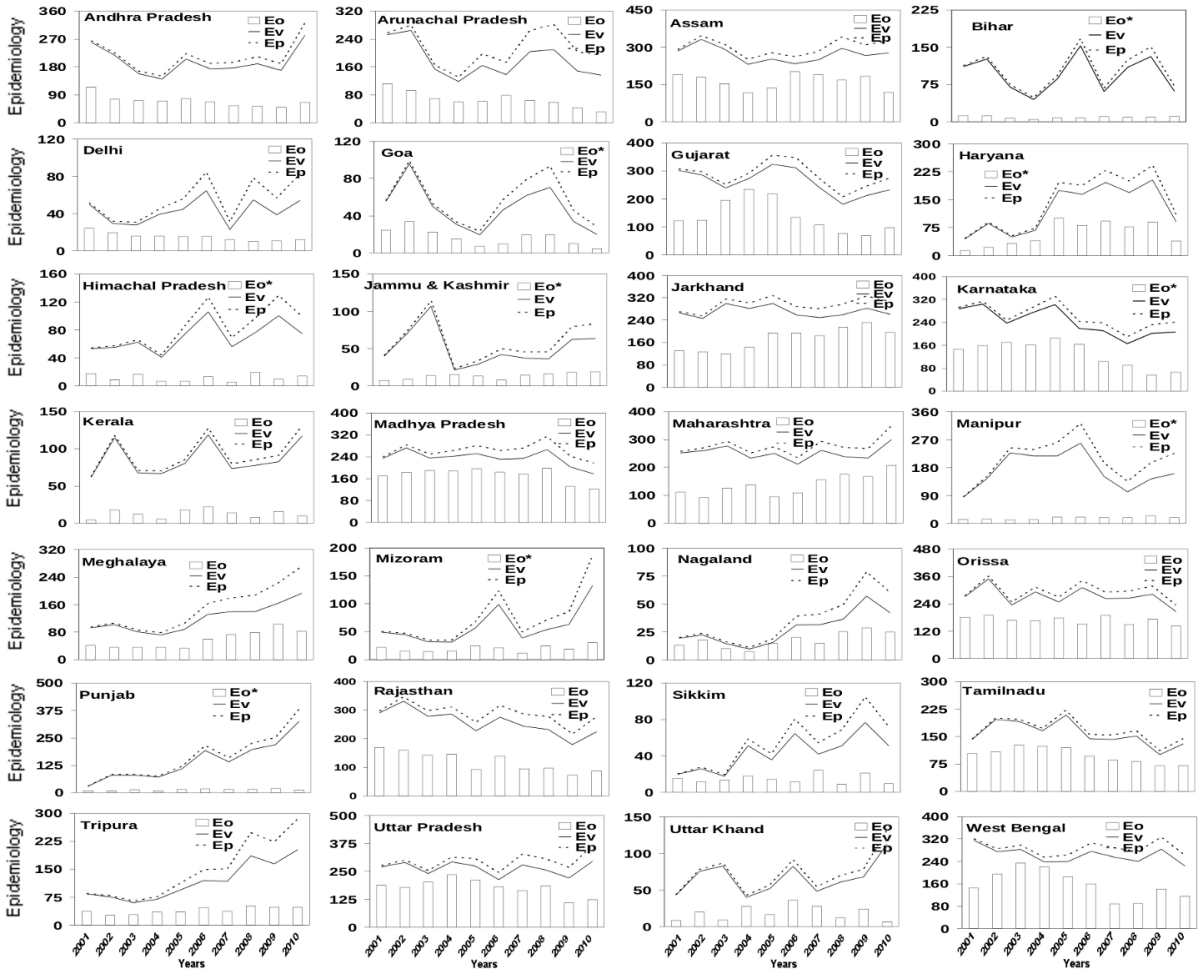
Comparison of observed annual epidemiology load (E_O_) with epidemiology load based on vector load (E_V_) calculated using days of vector genesis and constant human population with potential epidemiology load (E_P_) (growth in human population included) over 28 states of India. The annual epidemiology is calculated as the number of blood samples that positive. The days of vector genesis here represent days in a year that fulfill only meteorological condition of humidity for genesis of mosquitoes. The annual epidemiology has been scaled by a factor (500 for which marked * and rest with 1000, as indicated) for easy comparison.

To examine the relative roles of the three weather variables, vector load was first calculated with each of these three variables separately. With only temperature as the condition for mosquito genesis, the calculated number of malarial cases has very little correspondence with the number of reported cases (Figure 3); indeed, only five states are characterized by correlation coefficient between Eo and E_V_ significant at 99% confidence level for the degrees of freedom involved; the corresponding number for E_P_ is 8 (Table 2). Similar conclusions also hold for epidemiology load calculated with only rainfall as the condition for mosquito genesis (Figure 4), and only humidity as the condition for mosquito genesis (Figure 5); the numbers of states with correlation coefficient between Eo and either E_P_ or E_V_ significant at 99% confidence level are very few with either rainfall or humidity (Table 2).

**Figure 6:**
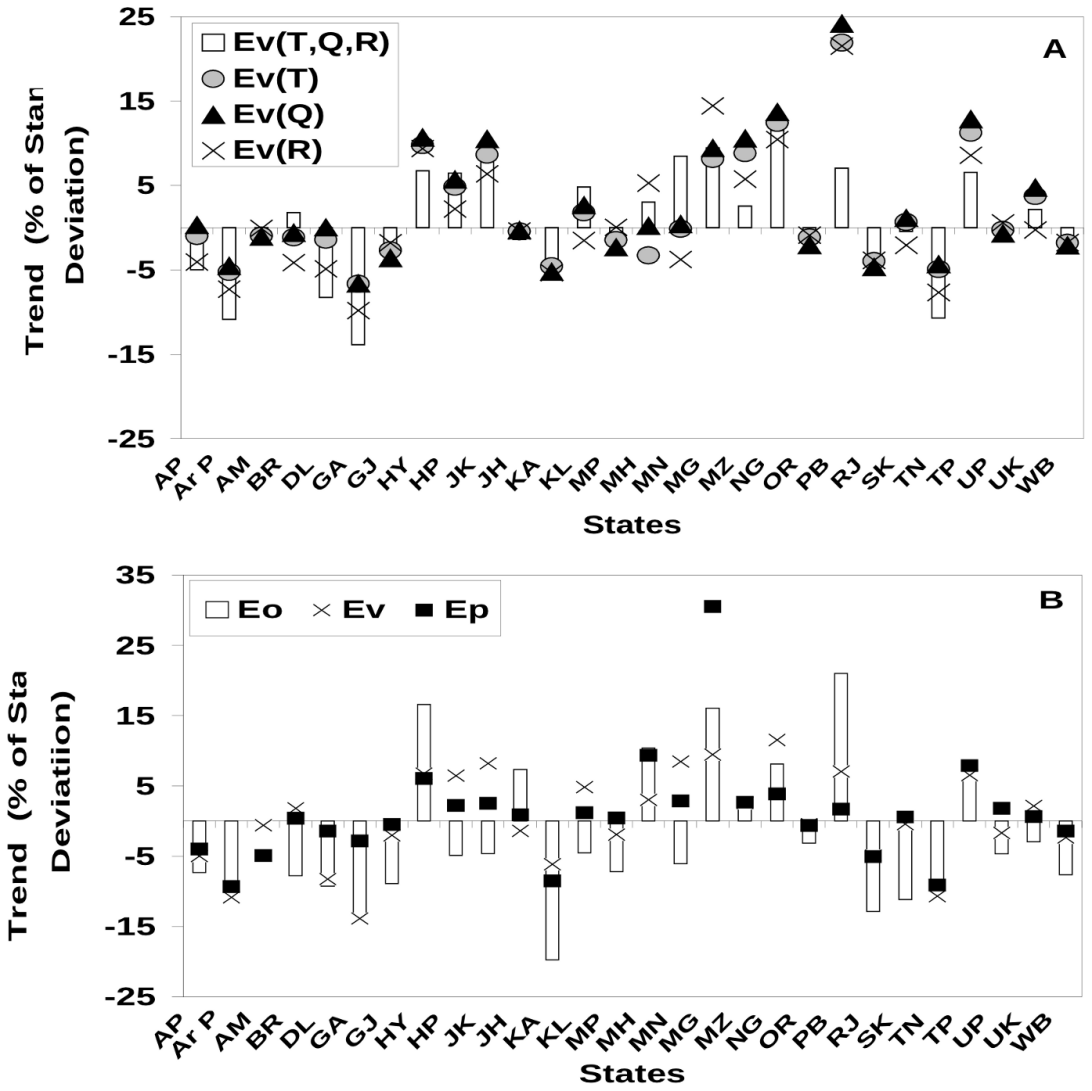
Linear trends in observed and calculated epidemiology (as % of respective standard deviation) (A) calculated based on genesis constraint of only temperature, only humidity, only rainfall and combined for the period of 1961–2010. (**B**) Observed epidemiology (E_O_), vector load (E_V_) and epidemiology potential (E_P_) for the 28 states based on data for the period 2001–2010.

It can be seen that the linear trends in the number of genesis days with individual weather variables can be quite different from those with all the three weather variables (Figure 6, top panel); in particular, the trends with the combined criteria can be either larger or smaller than those with a single weather variable. For easy assessment of significance, all trends have been expressed as percentage of respective standard deviation. More importantly, both appreciable positive and negative trends are present across the states (Figure 6, top panel). The linear trends calculated with either Eo and E_P_, match well with those for observed number of malaria cases for most states (Figure 6, bottom panel). There is no significant or systematic improvement in the calculated trends against the observed trends, indicating the number of genesis days as the driving factor for the changes in malaria cases. There are only four states, Jharkhand (JH), Jammu and Kashmir (JK), Meghalaya (MG), and Maharashtra (MH), for which the trends are in opposite direction, but with marginal difference; out of these four states, the first three are mountainous. It is noteworthy; however, that inclusion of change in human population often reduces the trends in calculated malaria cases (Figure 6, bottom panel). While many states show decline in the number of genesis days and corresponding decline in malaria load, a few states show significant positive trends, notably Maharashtra (MH) and Tripura (TP) (Figure 6, bottom panel). A few states, notably Himachal Pradesh (HP), Rajasthan (RJ) and Meghalaya (MG), show steep rise in both malaria prevalence and genesis days in recent years, indicating, once again, the driving role of the weather variables.

## Discussions and Conclusions

The relative roles of weather variables in vector genesis, and increase in exposure due to growth in human population, are critical inputs for many issues like effectiveness of vector control and assessment of impact of climate change. Our results show that vector load defined in terms of genesis days calculated from the combined daily values of temperature, rainfall and humidity provides a close association with malaria; none of the weather variables alone provides significant skill.

The present results, along with our earlier results on Arunachal Pradesh [37], show that the association between weather variables and malaria is robust in terms of applicability to diverse climatic regions; the 28 states of India represent climates from the tropical to the higher latitudes and regions of varying endemicity. Another important insight is the delineation of the role of weather variables from the impact of human population in the disease occurrence; our results show the weather variables to be the primary drivers.

Death due to malaria depends on a host of processes like vector control, quality of health services, land management as well as public awareness. However, identification of factors responsible for the vector dynamics can reduce the uncertainties in designing control and mitigation strategies. Our identification of the strong association between vector loads computed based on combined conditions of weather variables at daily scale provides a basis for forecasting malaria outbreak at short time scales by using high resolution weather monitoring and forecasts. The present results can enable methodology for identification of the peaks in vector population which would precede the disease outbreak by a typical incubation time of the parasite in the human host [37]. It is worth emphasizing that the toll of the epidemic, in terms of deaths, will also depend critically on several other factors like access to health care, especially in remote areas [41]. To further quantify the association between vector load and disease prevalence it is necessary to calibrate the parameters for each region through extensive observational programs.

In the same way that we have considered trends in annual loads, it is possible to examine trends in seasonal or even daily vector loads. A primary requirement for such an analysis is reliable malaria data at seasonal/daily scales over each state. Similarly, availability of systematic malaria data over smaller spatial units (like districts) can be used for examining trends at higher spatial resolution. However, as the changes in climate as well as in population are slow processes, analysis at annual scale is logical.

The calculation of days of vector genesis in the present version is based on daily values of the meteorological variables. It is desirable, and possible, to include episodic (say 3-day window) values of the variables to include effects of the preceding meteorological conditions on vector genesis. This would ensure that flooding due to rains before genesis day can also affect the survival of the larvae. Similar arguments are also valid for extreme temperature and dryness. However, we have not included these effects here explicitly for simplicity; our results based on only the one-day criteria may be further improved due to multi-day (episode) based criteria. Similarly, we have avoided detail effects of the meteorological variables on the life cycle of mosquito. For example, the effect of rainfall on mosquito dynamics is likely to be more at the larval stage. However, since reduction in the number of larvae due to (flushing by) rainfall is likely to reduce the number of adult mosquitoes, we have not separated the differential effect at different stages of the mosquito life cycle.

The results bring out clearly the minimum necessary analysis for investigating impact of climate change on malaria. At the same time, close relation between vector genesis days and the disease provides an excellent tool for investigating impacts of regional climate change on malaria, especially to investigate impact scenarios where the change, rather than the actual malaria load cases, is more important. In addition to genesis, there are other processes that may become important in situations of extreme climate change. For example, the life cycle of the malaria parasite inside the mosquito and the human host is important for transmission. Large changes in temperature can lead to reduction in the duration of gonotrophic cycle and in the extrinsic incubation period of malaria parasite and affect the rate of transmission [42]. Similarly, possible changes in the immunology profile of a people due to factors like migration may also become important in the long term. However, most of the processes in the post-larval stages become less important if vector sanitation is carried out at the genesis sites. The framework and the methodology are quite generic and can be applied in any geographical location with required calibration.

In practice, several species of mosquito are responsible for malaria in India [43]–[46]. The genesis and survival criteria may vary somewhat from species to species; similarly, the effects of local environmental conditions like run-off, land use and water stagnation may affect mosquito dynamics. However, as our results show, these are likely to be secondary effects.

The two parameters that define our model, α_v_ (k) and α_p_ (k), characterize various local factors (for a state) that modulate vector dynamics as well as malaria load. While we have considered a single value each for α_p_ and α_v_, it is possible, and desirable, to consider values α_p_ and α_v_ that represent characteristics of a state. However, this may not change our results qualitatively, as it would only mean changing the values of α_p_ and α_v_ to calculate Ev and Ep for each state, while increasing the complexity of the model. For practical application, it would be necessary to determine the parameters like Ep for different states and different seasons. As discussed in our earlier work [37], this is possible by considering climatological conditions as well as factors like population and seasonablity of outdoor activity (exposure). The methodology for such an extension is, however, straight forward.
